# Adaptive Optimization of Vascular-Targeted Photodynamic Therapy Efficiency Based on Hyperspectral-Photoacoustic Dual-Modality Imaging Feedback

**DOI:** 10.34133/bmef.0225

**Published:** 2026-02-27

**Authors:** Rongrui Zhang, Jingrui Zhao, Shasha Wang, Jing Lv, Junduo Liu, Jing Liu, Yawen Wang, Lei Fu, Weihui Zeng, Qiangzhou Rong, Cuiping Yao

**Affiliations:** ^1^ Key Laboratory of Biomedical Information Engineering of Ministry of Education, Institute of Biomedical Photonics and Sensing, School of Life Science and Technology, Xi’an Jiaotong University, Xi’an 710049, China.; ^2^ Department of Dermatology, The Second Affiliated Hospital of Xi’an Jiaotong University, Xi’an 710004, China.

## Abstract

**Objective:** To enhance vascular-targeted photodynamic therapy (V-PDT) efficacy by integrating real-time dosimetric monitoring and adaptive irradiance modulation based on dynamic physiological feedback. **Impact Statement:** This study presents a closed-loop, dual-modality optical imaging-guided V-PDT platform that enables individualized, oxygen-informed irradiance control, improving therapeutic precision and efficiency. **Introduction:** While V-PDT is a promising, minimally invasive treatment for tumors and vascular abnormalities, its efficacy is often hindered by rapid oxygen depletion under high irradiance, leading to treatment-limiting hypoxia. Accurate, real-time assessment of both photosensitizer concentration and blood oxygenation is essential to guide optimized therapeutic strategies, yet such capability has remained elusive in clinical settings. **Methods:** We developed a dual-modality imaging system integrating hyperspectral imaging (HSI) and optical-resolution photoacoustic microscopy (OR-PAM). HSI provides real-time, quantitative mapping of blood oxygen saturation and photosensitizer concentration, and OR-PAM provides high-resolution structural imaging of vascular networks. A personalized V-PDT protocol was implemented, where light irradiance was dynamically modulated in response to real-time blood oxygen feedback. **Results:** Real-time imaging confirmed that dynamic irradiance modulation effectively suppressed treatment-induced hypoxia while preserving therapeutic oxygen availability. The personalized-irradiation protocol significantly improved therapeutic efficacy compared with conventional fixed-irradiance protocols under identical photosensitizer dosage conditions. PAM-based structural analysis further showed that vascular damage strongly correlated with oxygen-informed irradiance adjustments. **Conclusion:** By integrating real-time dosimetry monitoring and feedback-controlled illumination, this study presents a closed-loop V-PDT strategy that overcomes oxygen depletion, enabling precise and efficient therapy tailored to individual tissue responses.

## Introduction

Photodynamic therapy (PDT) is a nonthermal therapeutic modality that employs the coordinated action of a photosensitizer, specific-wavelength light, and molecular oxygen to induce cellular damage or death. It has been widely applied in the treatment of tumors, vascular abnormalities, and infectious diseases [1–4]. The core mechanism of PDT involves the activation of the photosensitizer by light irradiation, leading to the generation of reactive oxygen species (ROS), primarily singlet oxygen (^1^O₂), which subsequently damages targeted cells. In recent years, PDT has emerged as the fourth major minimally invasive treatment modality, following surgery, radiotherapy, and chemotherapy.

Vascular-targeted PDT (V-PDT) has emerged as a specialized PDT modality designed to selectively damage pathological blood vessels. In V-PDT, photosensitizers preferentially accumulate within the vasculature, and their light-induced activation leads to vascular injury, reduced perfusion, or even complete vessel occlusion. By disrupting blood supply to diseased tissue, V-PDT has demonstrated substantial promise in the treatment of vascular malformations, as well as certain solid tumors such as prostate cancer [5–11]. Importantly, because its therapeutic outcome is tightly coupled to vascular physiology—particularly blood oxygen availability and consumption—V-PDT provides an ideal model for investigating oxygen-dependent PDT mechanisms and for developing optimized, dosimetry-guided illumination strategies.

Most clinically used photosensitizers mediate type II PDT, an oxygen-dependent process in which molecular oxygen participates directly in photochemical reaction kinetics, thereby determining the yield of ^1^O₂ and ultimately the therapeutic efficacy of PDT [12–15]. Consequently, tissue oxygen availability becomes a critical factor influencing treatment outcomes. However, rapid oxygen depletion caused by photochemical reactions and PDT-induced vascular occlusion often leads to hypoxia within the treatment region, significantly compromising therapeutic effectiveness. To address this issue, current research efforts have primarily focused on enhancing oxygen supply or reducing oxygen consumption during illumination, with the aim of maintaining adequate tissue oxygenation and improving therapeutic efficacy [16–19].

Oxygen carriers, known for their high oxygen storage capacity, are among the most commonly used strategies to enhance oxygen supply during PDT [20–25]. These carriers are typically derived from natural substances such as hemoglobin and function by physically adsorbing oxygen molecules and transporting them to the targeted lesion site, thereby ensuring adequate oxygen availability for photosensitization. In addition to oxygen carriers, certain chemical agents can be delivered to the target tissue, where they undergo in situ chemical reactions to generate oxygen and locally elevate oxygen tension [26–29]. Although strategies such as exogenous oxygen delivery and chemical oxygen generation can temporarily alleviate hypoxia, they are typically limited by rapid oxygen-release kinetics, poor tissue penetration, and a lack of sustained oxygen delivery throughout the treatment period. Moreover, existing treatment regimens lack the flexibility to be personalized according to individual patient conditions and are devoid of dynamic feedback mechanisms.

Under high irradiance, excessive consumption of molecular oxygen outpaces the tissue’s ability to replenish it, resulting in diminished therapeutic efficacy [30]. Optimizing the irradiation protocol to reduce oxygen consumption represents one of the most cost-effective strategies for sustaining tissue oxygenation during PDT [31–34]. Under a constant total light dose, reducing the irradiance or employing fractionated/metronomic illumination can slow down photochemical oxygen consumption, thereby maintaining tissue oxygenation at relatively high levels and enhancing ^1^O₂ generation as well as overall therapeutic efficacy [6,35–38]. However, such low-irradiance protocols substantially prolong the treatment duration, thereby increasing the burden on both patients and clinical resources. Although various approaches have been explored for oxygen monitoring during PDT, most studies have primarily focused on characterizing the oxygen dynamics under conventional high-irradiance illumination conditions [39–41]. These methods typically emphasize real-time tracking of tissue oxygenation to better understand therapy-induced hypoxia, yet they have rarely translated these measurements into direct optimization of illumination parameters. In particular, the potential benefits of low-irradiance, prolonged illumination have rarely been experimentally validated using quantitative real-time imaging data. Therefore, there is an urgent need for a personalized treatment strategy capable of dynamically adjusting irradiance in response to real-time tissue oxygenation levels. Such an approach would help achieve a balance between oxygen consumption and supply, ultimately enhancing the overall efficiency of PDT.

Herein, we propose a dual-modality imaging system that integrates hyperspectral imaging (HSI) and optical-resolution photoacoustic microscopy (OR-PAM). The system enables real-time monitoring of key V-PDT dosimetric parameters, including photosensitizer concentration and blood oxygen saturation (sO₂), via HSI, while PAM provides high-resolution imaging and quantitative analysis of vascular structural changes before and after treatment, allowing for structured evaluation of therapeutic efficacy. Based on this system, we further introduce a personalized light delivery strategy in which irradiance is dynamically adjusted according to real-time blood oxygen levels. By cyclically modulating irradiance in response to oxygenation changes, this approach effectively regulates oxygen consumption rates and improves treatment efficiency. This strategy overcomes the inherent limitations of conventional fixed-irradiance protocols, which often fail to balance oxygen demand and supply during therapy. Experimental results demonstrate that, under consistent photosensitizer dosage, the proposed method significantly enhances therapeutic outcomes while reducing total treatment time. In summary, this study proposes and validates a closed-loop optimization framework for V-PDT comprising dosimetry monitoring, light modulation, and therapeutic evaluation, and highlights the potential of the integrated HSI–PAM platform in enabling personalized and precise V-PDT.

## Results

To quantitatively compare blood oxygenation dynamics among the 3 treatment groups, we defined 3 physiologically meaningful metrics derived from the sO₂ time courses: oxygen depletion rate (*K*_sO₂_, %/min), time-to-hypoxia (*T*_hypoxia_, s), and oxygen recovery amplitude (*ΔsO₂*, %). In addition to oxygenation metrics, the vascular shrinkage ratio (*VSR*, %) was used to quantitatively characterize PDT-induced vascular damage based on structural changes detected by OR-PAM. Together, these parameters provide an objective and reproducible assessment of oxygen consumption, reoxygenation, and microvascular response during and after V-PDT, with detailed definitions provided in Materials and Methods.

**Fig. 1.**
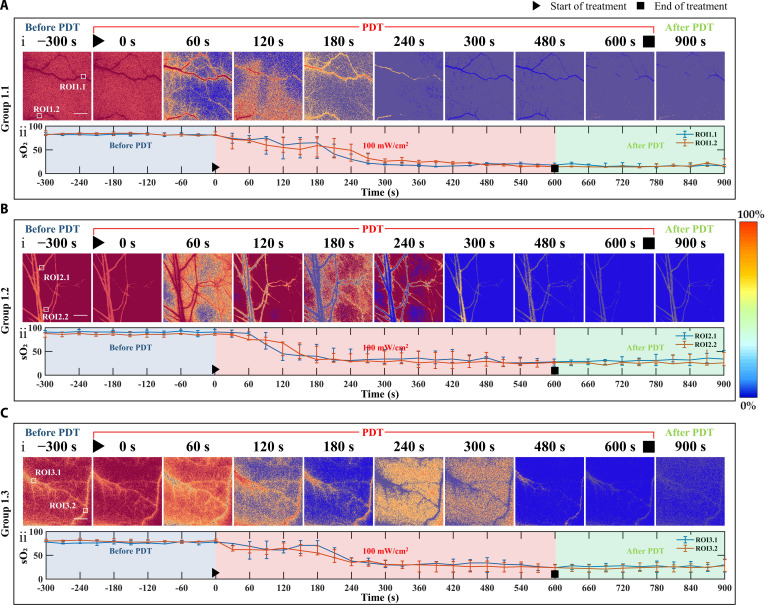
Representative results of sO2 dynamics in the high-irradiance short-duration treatment group (scale bar, 1 mm). (A to C) Three individual mice from the high-irradiance group, continuously irradiated at 100 mW/cm^2^ for 10 min: (i) 2-dimensional variation of blood oxygen in the target blood vessels at different time points before, during, and after treatment; (ii) sO2 time courses extracted from different ROIs, where the blue segment corresponds to pretreatment oxygen saturation, the red segment denotes changes during the 100 mW/cm^2^ irradiation, and the green segment indicates post-treatment recovery.

**Fig. 2.**
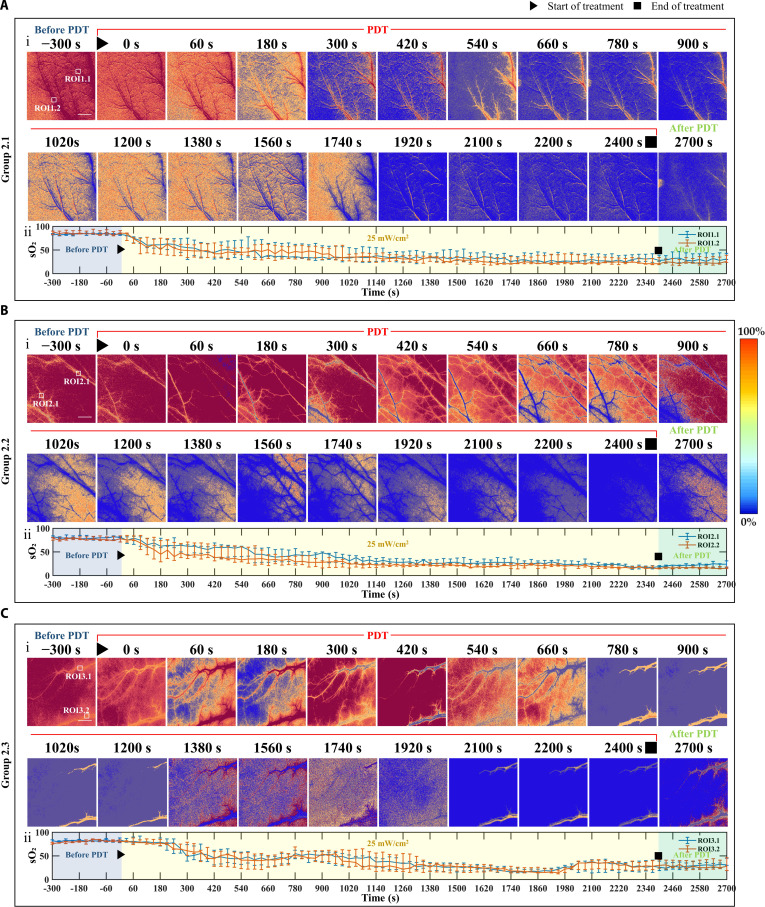
Representative results of sO_2_ dynamics in the low-irradiance long-duration treatment group (scale bar, 1 mm). (A to C) Three individual mice from the low-irradiance group, continuously irradiated at 25 mW/cm^2^ for 40 min: (i) 2-dimensional variation of blood oxygen in the target blood vessels at different time points before, during, and after treatment; (ii) sO_2_ time courses extracted from different ROIs, where the blue segment indicates pretreatment oxygen saturation, the yellow segment denotes the changes during the 25 mW/cm^2^ irradiation, and the green segment shows the post-treatment recovery.

**Fig. 3.**
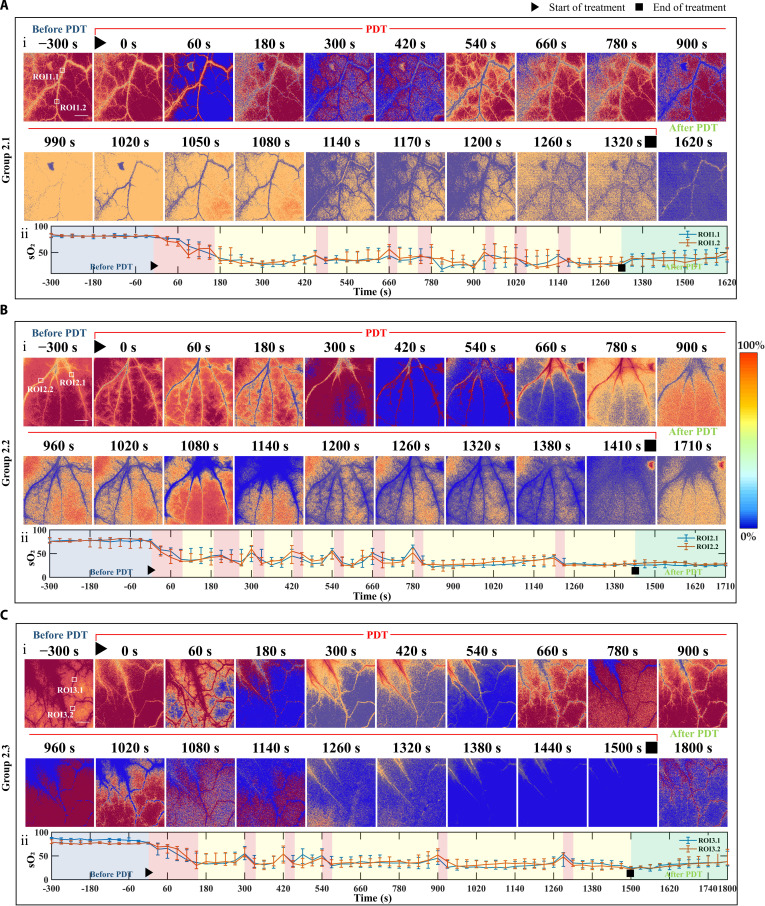
Personalized-irradiation strategy guided by real-time sO_2_ levels during V-PDT (scale bar, 1 mm). (A to C) Three individual mice from the personalized-irradiation group, where the irradiance alternates between 100 and 25 mW/cm^2^ based on real-time sO_2_ feedback: (i) 2-dimensional variation of blood oxygen in the target blood vessels at different time points before, during, and after treatment; (ii) sO_2_ time courses obtained from different ROIs, with the blue segment indicating pretreatment oxygen saturation, the red segment representing changes during the 100 mW/cm^2^ irradiation, the yellow segment representing changes during the 25 mW/cm^2^ irradiation, and the green segment showing the post-treatment recovery phase.

To evaluate the effects of different illumination protocols on oxygenation dynamics, a total of 30 mice were randomly assigned to 3 treatment groups (*n* = 10 per group): high-irradiance short-duration, low-irradiance long-duration, and personalized-irradiation. For clarity in visualization, Figs. 1 to 3 present representative results from 3 mice in each group, whereas all statistical analyses were performed using the full cohort of 10 mice per group. Table 1 presents the treatment parameters and quantitative vascular–oxygenation metrics for the 3 irradiation protocols. These metrics include oxygen depletion rate, time to hypoxia, oxygen recovery amplitude, and *VSR*, enabling a direct comparison of oxygen dynamics and treatment outcomes across groups. Detailed statistical analysis methods are provided in Materials and Methods.

**Table 1.** Summary of treatment parameters and quantitative vascular–oxygenation metrics for the 3 V-PDT irradiation protocols

V-PDT protocol	Average treatment duration (s)			Photosensitizer (μg/ml)	*K*_sO2_ (%/min)	*T*_hypoxia_ (s)	*ΔsO2* (%)	*VSR* (%)
	100 mW/cm^2^	25 mW/cm^2^	Total					
100 mW/cm^2^ continuous	600	0	600	65.39 ± 0.42	10.36 ± 1.56	280 ± 72	3.70 ± 2.11	22.47 ± 9.97
25 mW/cm^2^ continuous	0	2,400	2,400	65.15 ± 0.86	2.82 ± 0.48	1,530 ± 220	16.60 ± 7.55	52.53 ± 17.73
Personalized irradiation	330 ± 30	1,080 ± 120	1,410 ± 90	65.72 ± 1.28	3.71 ± 0.55	1,020 ± 310	13.70 ± 6.96	57.63 ± 14.73

### Differences in blood oxygenation dynamics

As shown in Fig. 1, representative results from 3 mice subjected to high-irradiance short-duration treatment (100 mW/cm^2^, continuous illumination for 10 min) are presented. The figure quantitatively illustrates the 2-dimensional distribution of sO₂ in the target vasculature within the dorsal skinfold window before treatment (blue region), during treatment (red region), and after treatment (green region). Corresponding sO₂ time courses from each region of interest (ROI) are also displayed. The spatial sO₂ maps were processed using pseudocolor rendering.

To quantitatively assess sO₂ changes in each treatment group, 2 ROIs, each measuring 30 × 30 pixels, were selected per group. The average sO₂ value of all pixels within each ROI at a given time point was calculated as the representative oxygenation level. As shown in Fig. 1, in the high-irradiance group, blood oxygen saturation dropped rapidly at the onset of treatment and reached a minimum around 300 s, remaining low until the end of illumination. The stable, normal pretreatment sO₂ levels indicate that the window chamber implantation did not compromise vascular integrity or perfusion, ensuring that surgical procedures did not confound the treatment outcomes.

Figure 2 presents the quantitative analysis of sO₂ changes in the low-irradiance group (25 mW/cm^2^, continuous illumination for 40 min), showing pretreatment (blue region), during treatment (yellow region), and post-treatment (green region) oxygenation states. In this group, sO₂ also decreased immediately after illumination began; however, the oxygen depletion rate was substantially slower than that in the high-irradiance group (2.82 ± 0.48 versus 10.36 ± 1.56%/min, *P* < 0.0001). Furthermore, post-treatment reoxygenation was more pronounced, with an oxygen recovery amplitude of 16.60 ± 7.55%, significantly higher than that of the high-irradiance group (3.70 ± 2.11%, *P* < 0.001). Together, Figs. 1 and 2 demonstrate that, under the same total light dose, variations in irradiance intensity significantly influence the rate and extent of oxygen depletion during V-PDT.

In the personalized-irradiation group, real-time sO₂ was used to modulate irradiance, and the control threshold was set at 40% sO₂. This value was selected based on preliminary observations showing that sO₂ levels below ~40% resulted in persistent hypoxia with poor recovery, whereas maintaining sO₂ above this level allowed a better balance between oxygen consumption and vascular oxygen resupply. Previous microvascular oxygenation and PDT studies have shown that sO₂ levels in the range of 30% to 40% represent a physiologically meaningful transition point marking the onset of functional hypoxia—where oxygen delivery becomes insufficient to meet metabolic demand, oxygen extraction efficiency declines sharply, and photochemical ^1^O₂ generation is substantially reduced [42]. Furthermore, classical PDT studies indicate that when intravascular oxygenation falls into the 30% to 40% range, the photochemical generation of singlet oxygen becomes significantly impaired [43]. These reports support the use of a 40% threshold as a physiologically meaningful boundary to prevent deep treatment-limiting hypoxia while preserving photodynamic efficacy. Therefore, 40% was chosen as a pragmatic compromise threshold for this proof-of-concept study.

Figure 3 illustrates the sO₂ dynamics during the personalized-irradiation protocol guided by real-time sO₂ levels. The initial irradiance was set at 100 mW/cm^2^. Based on the monitored sO₂ values, the system was programmed to automatically switch to a lower irradiance of 25 mW/cm^2^ when sO₂ dropped below 40%. Once sO₂ recovered above 40%, the system switched back to the high irradiance of 100 mW/cm^2^. This periodic modulation of irradiance continued until the cumulative light dose reached 60 J/cm^2^, at which point the treatment was terminated. As shown in Fig. 3, blood oxygenation exhibited notable recovery during low-irradiance intervals. In contrast, sO₂ levels decreased rapidly and rarely recovered during high-irradiance phases. Moreover, the ability of blood vessels to restore oxygenation during the low-irradiance periods declined progressively as the total treatment time increased.

As summarized in Table 1, the oxygen depletion rate was significantly higher in the high-irradiance group (10.36 ± 1.56%/min) than in the low-irradiance group (2.82 ± 0.48%/min) and the personalized-irradiation group [3.71 ± 0.55%/min; one-way analysis of variance (ANOVA), *P* < 0.001; Tukey’s post hoc test, *P* < 0.001 for high versus low and high versus personalized]. Time-to-hypoxia was shortest in the high-irradiance group (280 ± 72 s), longest in the low-irradiance group (1,530 ± 220 s), and intermediate in the personalized-irradiation group (1,020 ± 310 s; Tukey’s post hoc test, *P* < 0.001 for all pairwise comparisons). Oxygen recovery amplitude after treatment was also significantly smaller in the high-irradiance group (3.70 ± 2.11%) than in the low-irradiance group (16.60 ± 7.55%, *P* < 0.001) and the personalized-irradiation group (13.70 ± 6.96%, *P* = 0.003; Tukey’s post hoc test).

To further determine which groups differed significantly, Tukey’s HSD post hoc test was performed following one-way ANOVA. Pairwise comparisons showed that the high-irradiance group exhibited a significantly higher oxygen depletion rate than both the low-irradiance group and the personalized-irradiation group (Tukey’s post hoc test, *P* < 0.001 for both comparisons), whereas the difference between the low-irradiance group and the personalized-irradiation group was not statistically significant (*P* = 0.064). For time-to-hypoxia, all 3 groups were significantly different from one another (Tukey’s post hoc test, *P* < 0.001 for all pairwise comparisons), confirming that the low-irradiance protocol delays the onset of hypoxia and that the personalized-irradiation protocol lies between the 2 fixed-dose conditions. For oxygen recovery amplitude, both the low-irradiance group and the personalized-irradiation group showed significantly greater post-treatment oxygen recovery than the high-irradiance group (Tukey’s post hoc test, *P* < 0.01), while the difference between the low-irradiance group and the personalized-irradiation group was not significant. Regarding *VSR*, both the low-irradiance and personalized-irradiation protocols induced significantly greater vascular shrinkage than the high-irradiance protocol (*P* < 0.001, Tukey’s post hoc test), with no significant difference between the low-irradiance group and the personalized-irradiation group (*P* = 0.76).

### Differences in therapeutic efficacy

**Fig. 4.**
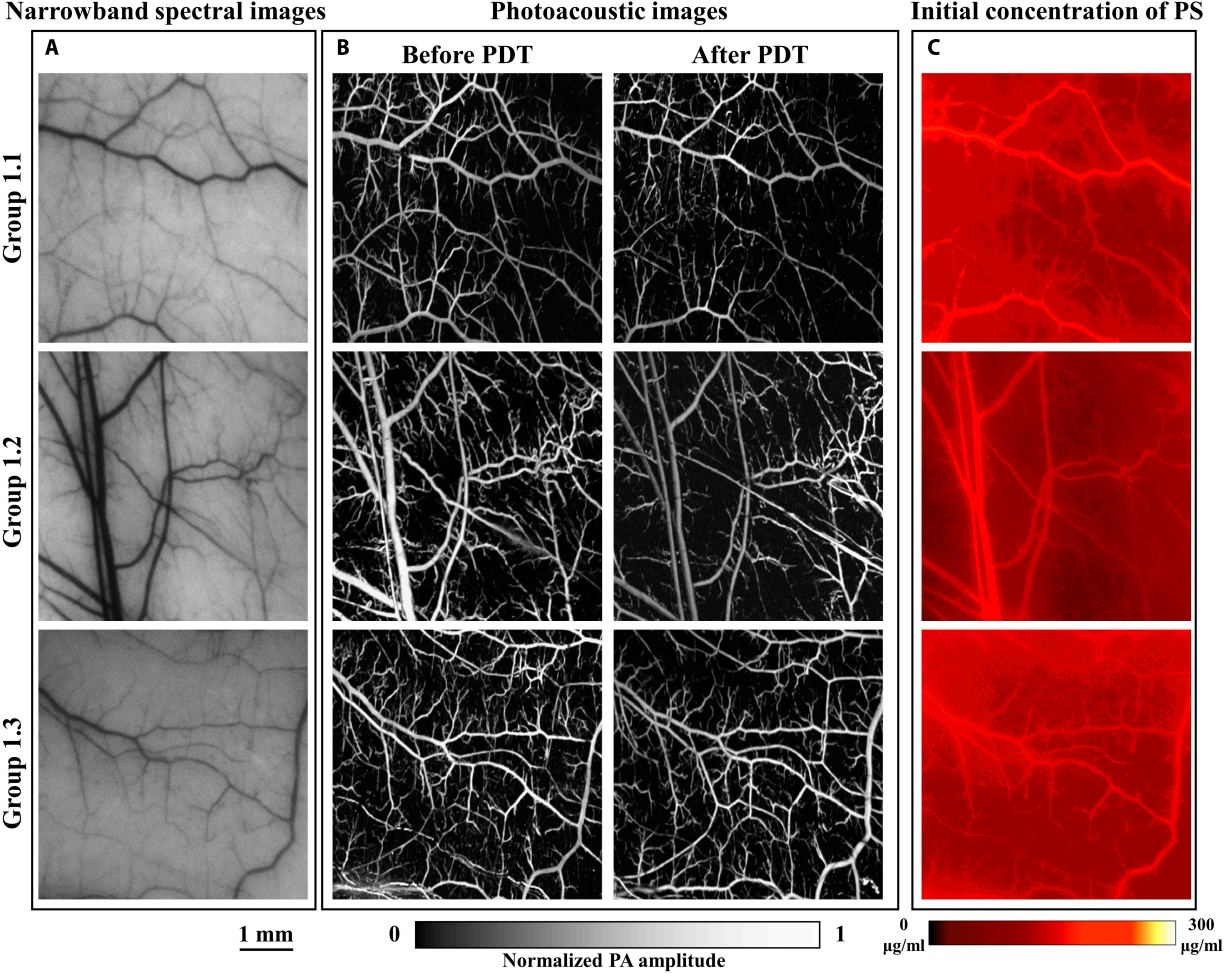
High-irradiance short-duration group V-PDT results (scale bar, 1 mm). (A) Narrowband spectral image at 550 nm of the target blood vessels. (B) Comparison of OR-PAM images of the target vessels before and after treatment. (C) Initial distribution of photosensitizer concentration.

**Fig. 5.**
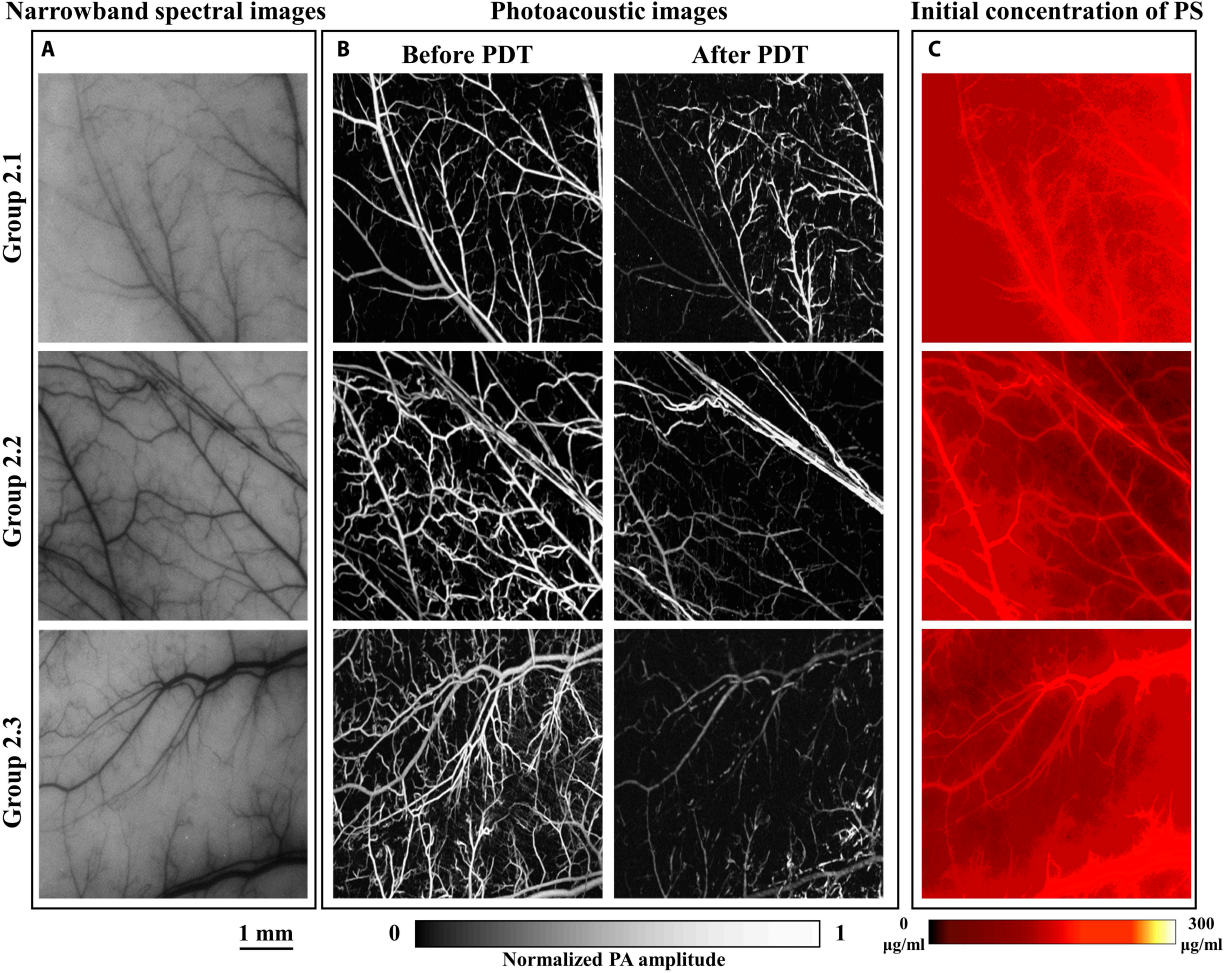
Low-irradiance long-duration group V-PDT results (scale bar, 1 mm). (A) Narrowband spectral image at 550 nm of the target blood vessels. (B) Comparison of OR-PAM images of the target vessels before and after treatment. (C) Initial distribution of photosensitizer concentration.

**Fig. 6.**
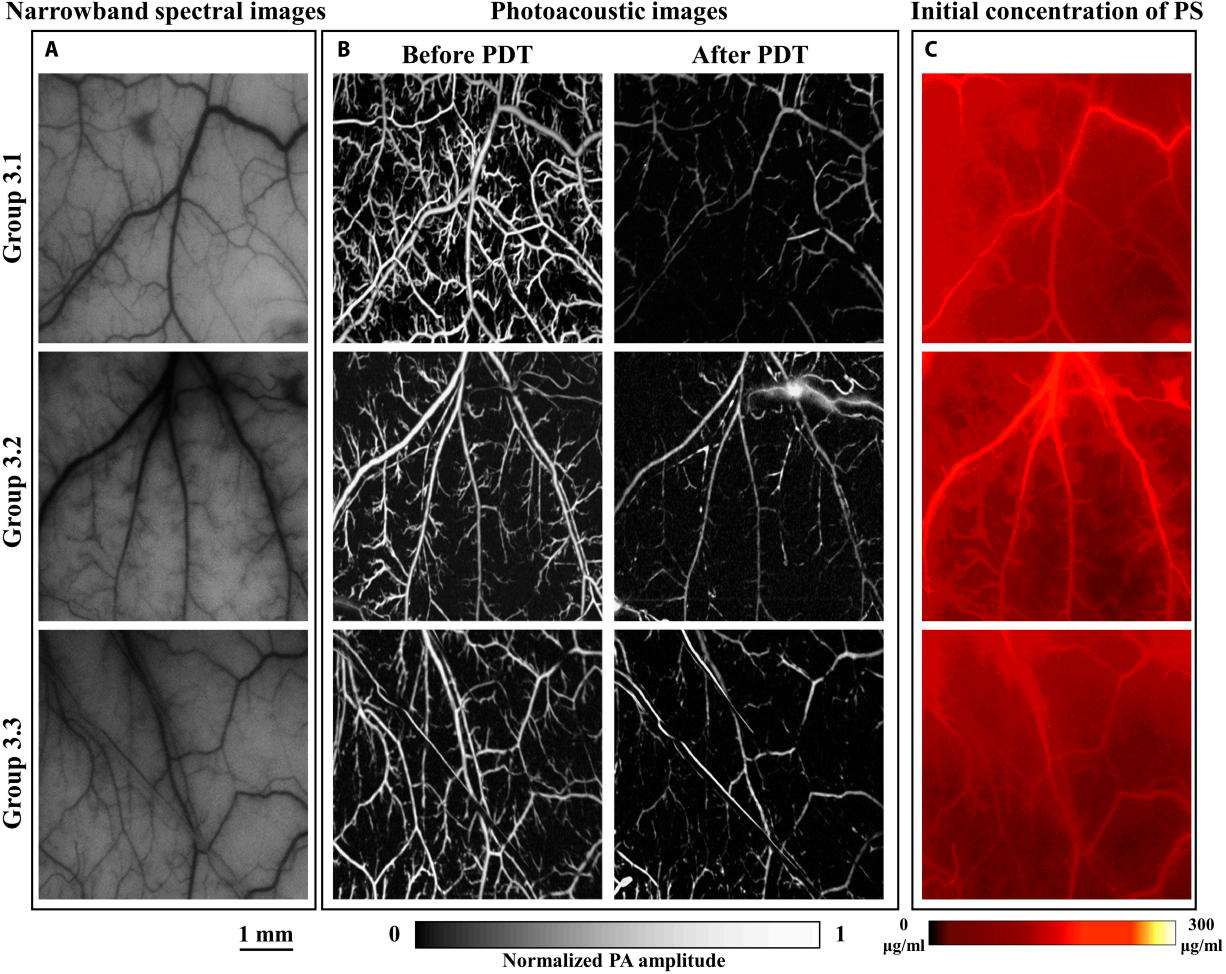
Personalized-irradiation group V-PDT results (scale bar, 1 mm). (A) Narrowband spectral image at 550 nm of the target blood vessels. (B) Comparison of OR-PAM images of the target vessels before and after treatment. (C) Initial distribution of photosensitizer concentration.

Figures 4 to 6 show the results of V-PDT in the high-irradiance group, low-irradiance group, and personalized-irradiation group, respectively. Each figure presents the narrowband spectral images at 550 nm of the dorsal skinfold window vasculature before treatment, the initial photosensitizer concentration images, and the PAM of vascular changes before and after treatment.

The concentration of photosensitizer is a key factor in V-PDT. Although each mouse in the experimental groups was administered by the same dose of photosensitizer via tail vein injection, significant individual variation and potential errors during the animal model preparation may result in differences in photosensitizer content within the target vasculature. To minimize the impact of these differences on treatment outcomes, photosensitizer concentration in the dorsal skinfold window vasculature was measured after 1 min post-injection. Mice with significant deviations in photosensitizer concentration from the group average were excluded. The average concentration of photosensitizer in each treatment group was maintained at approximately 65 μg/ml.

As shown in Table 1, the blood oxygenation-guided personalized-irradiation protocol achieved favorable therapeutic outcomes. Consistent with the oxygenation dynamics, the *VSR* differed significantly among the 3 groups (one-way ANOVA, *P* < 0.001). Both the low-irradiance protocol and the personalized-irradiation protocol produced markedly greater vascular damage (52.53 ± 17.73% and 57.63 ± 14.73%, respectively) than the high-irradiance protocol (22.47 ± 9.97%; Tukey’s post hoc test, *P* < 0.001 for both comparisons), whereas no significant difference was observed between the low-irradiance group and the personalized-irradiation group (Tukey’s post hoc test, *P* = 0.76). Although the treatment duration was extended by 135% compared with the traditional high-irradiance short-duration protocol, the therapeutic efficacy, measured by the *VSR*, increased by 156.5%. Relative to the low-irradiance long-duration protocol, the personalized-irradiation protocol achieved comparable efficacy while reducing treatment time by approximately 41.25%. Therefore, real-time monitoring of blood oxygenation to guide personalized irradiance modulation in V-PDT may be more suitable for clinical application.

## Discussion

Changes in irradiance during V-PDT can effectively modulate variations in blood oxygen saturation. During V-PDT, the photochemical reaction of the photosensitizer and the formation of microvascular thrombosis consume molecular oxygen, resulting in a rapid decline in oxygen concentration in the target vasculature at the onset of treatment.

In high-irradiance treatments, sO₂ in the target vasculature decreases rapidly and continuously. This may cause the rate of oxygen consumption to exceed the rate at which oxygen is replenished through vascular diffusion, thereby reducing the efficacy of photodynamic damage. Conversely, in low-irradiance treatments, the rate of photochemical reaction is reduced, slowing oxygen consumption and providing sufficient time for oxygen replenishment, which enhances the efficacy of photodynamic damage. Although low irradiance increases V-PDT efficacy, it requires longer treatment times, thus increasing time costs for both clinicians and patients in a clinical setting.

The personalized-irradiation strategy guided by real-time blood oxygen feedback offers an effective solution for overcoming oxygen depletion during V-PDT. By dynamically adjusting the irradiance according to moment-to-moment oxygen levels in the target vasculature, this approach optimizes oxygen utilization during treatment. When oxygen saturation is high, the irradiance is increased to accelerate photochemical reactions; when oxygen levels drop, the irradiance is reduced, allowing sufficient time for vascular oxygen replenishment. This oxygen-informed modulation helps maintain an appropriate balance between oxygen consumption and supply. As a result, the blood oxygen-guided personalized-irradiation strategy not only shortens the treatment duration relative to conventional low-irradiance protocols but also achieves substantially higher therapeutic efficacy compared with high-irradiance, short-duration illumination.

Based on the optical absorption properties of hemoglobin, both types of hemoglobin exhibit strong absorption at 550 nm. Therefore, the 550-nm narrowband images can distinguish the main blood vessels and, to some extent, characterize V-PDT-induced vascular damage. Figures 4 to 6 compare the narrowband spectral images at 550 nm and PAM of the target vasculature before treatment. However, PAM offers higher spatial resolution, reveals finer capillary morphology, and provides depth-sensitive information, thereby enabling more accurate quantification of vascular changes before and after treatment. Consequently, PAM is likely one of the better methods for quantifying and assessing V-PDT efficacy. The inherent capability of HSI to detect both narrowband absorption spectra and fluorescence spectra may provide an optimal method for monitoring V-PDT dosimetric parameters.

The selection of 40% sO₂ as the control threshold was empirical and guided by pilot observations and known physiological evidence indicating that sO₂ values below ~40% correspond to functional hypoxia and reduced PDT efficiency. Although not systematically optimized in this study, our pilot tests showed that lower thresholds permitted deeper hypoxia, whereas higher thresholds caused unnecessary irradiance switching. A systematic comparison of multiple thresholds will be an important direction for future work to further refine adaptive PDT strategies.

The current system provides valuable insights for preclinical PDT research; however, its clinical application requires further exploration. The personalized-irradiation protocol based on real-time oxygen feedback offers a significant advantage in preventing hypoxia and optimizing treatment efficacy. However, clinical integration faces challenges such as synchronizing real-time data with existing clinical workflows and accommodating patient-specific variations in oxygenation and photosensitizer uptake. To address these challenges, future clinical trials should focus on refining the personalized-irradiation protocol to account for patient-specific physiological variability and integrating real-time imaging with clinical decision-making. While the system holds great promise, further validation is needed to ensure its safe and effective use in clinical settings.

In summary, this study developed a dual-modality imaging system combining HSI and PAM. The system utilizes HSI for real-time monitoring of photosensitizer concentration and sO₂ changes during treatment, while PAM provides high-resolution imaging and quantitative analysis of vascular structural changes before and after treatment. Additionally, a personalized light delivery strategy was proposed, which dynamically adjusts irradiance based on blood oxygen levels, achieving a balance between oxygen consumption and supply and improving V-PDT efficacy. This research offers new insights into personalized V-PDT and advances the precision of V-PDT treatments.

## Materials and Methods

### HSI system

**Fig. 7.**
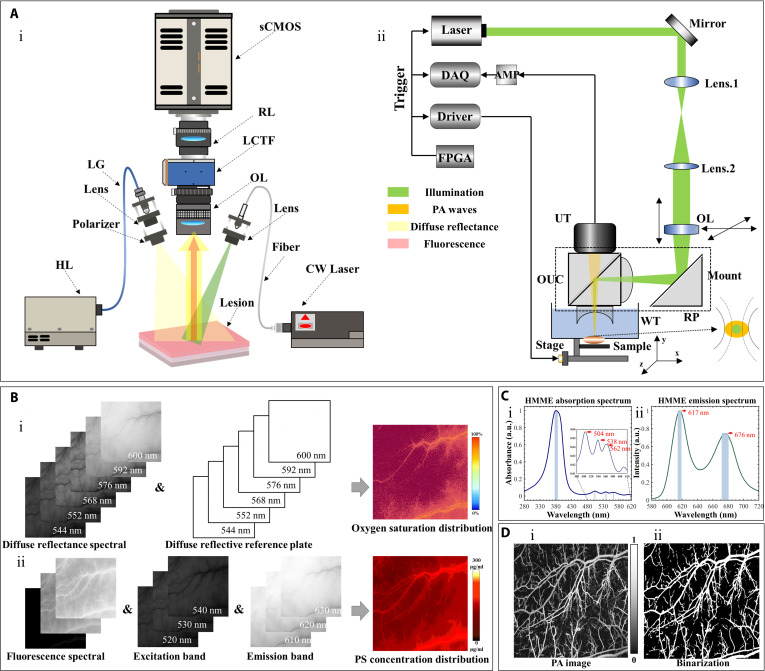
Schematic of the HSI and OR-PAM systems and the quantitative image reconstruction algorithms. (A) Hyperspectral and photoacoustic dual-modality imaging system: (i) HSI system schematic; (ii) OR-PAM system schematic. (B) Reconstruction algorithms for oxygen saturation and photosensitizer concentration: (i) spectral second-derivative algorithm for reconstructing blood oxygen saturation; (ii) dual-band normalization algorithm for photosensitizer concentration quantification. (C) Optical properties of HMME: (i) absorption spectrum of HMME; (ii) emission spectrum of HMME. (D) Vascular injury quantification: (i) raw vascular OR-PAM data; (ii) vascular binary image. RL, relay lens; LCTF, liquid-crystal tunable filter; LG, light guide; OL, objective lens; HL, halogen lamp; CW, continuous wave; DAQ, data acquisition card; AMP, amplifier; FPGA, field-programmable gate array; UT, ultrasonic transducer; OUC, photoacoustic combiner; WT, water tank; RP, reflecting prism.

As illustrated in Fig. 7A(i), the HSI system consists of an excitation laser (MGL-N-532, Changchun New Industries), a broadband halogen light source (GCI-060101, Daheng Optics), a liquid-crystal tunable filter (LCTF; VariSpec-VIS, PerkinElmer), and a scientific complementary metal-oxide semiconductor (sCMOS) camera (Zyla 4.2, Andor). The LCTF sequentially tunes wavelengths over a spectral range of 420 to 720 nm with a tuning step of 1 nm. The sCMOS camera enables high-SNR (signal-to-noise ratio) image acquisition at short exposure times, thereby reducing the overall HSI duration. The LCTF requires ~50 ms for wavelength tuning, and the camera exposure time is set to 100 ms. Complete hyperspectral acquisition over 420 to 720 nm with a 5-nm increment requires ~10 s under our standard imaging settings. However, the practical acquisition time may vary depending on the imaging task, such as whether only a subset of wavelengths is needed for sO₂ estimation or photosensitizer quantification. To facilitate system control, image acquisition, data storage, and processing, we developed custom software using LabView (National Instruments, Texas).

### Optical-resolution photoacoustic microscopy

As illustrated in Fig. 7A(ii), the OR-PAM system used in this study consists of a pulsed laser source, a photoacoustic probe, a motorized translation stage, a data acquisition unit, and a control module. A 532-nm pulsed laser (VPFL-G-20, Spectra-Physics) was used to excite the photoacoustic signal. The laser beam was first expanded by a lens group, then focused by an objective lens, and redirected by a right-angle prism into a custom-built high-sensitivity photoacoustic combiner. This combiner comprises an aluminum-coated prism and an uncoated prism, enabling coaxial alignment of optical and acoustic paths. The thin aluminum coating reflects the laser light while allowing acoustic signals to pass through. An optical correction lens mounted on the side of the combiner is used to correct optical aberrations introduced by the prism assembly.

The generated photoacoustic signals are acoustically focused by an acoustic lens. By adjusting the laser beam path and the position of the objective lens, optical and acoustic foci are precisely aligned to achieve high-sensitivity photoacoustic detection, which is then captured by an ultrasonic transducer (V214-BC-RM, Olympus). The right-angle reflecting prism, right-angle prism, correction lens, and acoustic lens are bonded together using ultraviolet-curable adhesive (NOA61 glue, Norland Products Inc.). The detected photoacoustic signals are amplified by 2 cascaded low-noise amplifiers (48 dB total gain, ZFL-500LN+, Mini-Circuits) and sampled at 250 MHz using a high-speed digitizer (ATS9350, AlazarTech). Raster scanning of the imaging target is performed by 2 orthogonal linear stages (PLS-85, Physik Instrument). The laser firing, stage movement, and data acquisition are all synchronized through a field-programmable gate array (FPGA)-based control system (myRIO, National Instruments).

For a 5 × 5 mm field of view (FOV), raster scanning was performed with a 5-μm fast-axis step size and a 10-μm slow-axis step size. This resulted in 1,000 A-lines per B-scan and 500 B-scans per volumetric image. With a 1-kHz laser repetition rate, each volumetric PAM image required approximately 8.3 min. PAM imaging was conducted before and after PDT to quantify vascular structural changes. We emphasize that vascular structural imaging is performed before and after PDT, not continuously during treatment, so this acquisition speed fully satisfies the study design. The axial resolution is determined by a combination of laser pulse width, acoustic attenuation in tissue, and the frequency response of the ultrasonic transducer. Based on the acoustic bandwidth of the high-frequency ultrasound transducer used in our OR-PAM system, the axial resolution is approximately 20 to 30 μm. The lateral resolution is primarily determined by the optical diffraction-limited focus of the 532-nm excitation beam and the numerical aperture of the objective lens, yielding an effective lateral resolution of ~5 μm. The 5-μm and 10-μm step sizes used for the fast and slow scanning axes, respectively, reflect the mechanical sampling intervals of the motorized stages and do not represent the intrinsic spatial resolution of the imaging system.

### Photosensitizer

The photosensitizer used in this study was hematoporphyrin monomethyl ether (HMME), a chemically stable compound known for its high singlet oxygen yield [44]. HMME has been widely employed in the clinical treatment of benign vascular malformations and related proliferative disorders [45]. As shown in Fig. 7C, its absorption and fluorescence emission spectra are presented [46].

### Animal model

Female BALB/c mice (25 to 30 g, 4 to 5 weeks old) were used in this study, obtained from the Laboratory Animal Center of Xi’an Jiaotong University. All procedures involving animals, including housing, experimental operations, and euthanasia, were conducted in accordance with ethical guidelines approved by the Experimental Animal Ethics Committee of Xi’an Jiaotong University.

To enable real-time monitoring of V-PDT dose-related parameters within target vasculature, a dorsal skinfold window chamber (DSWC) model was established in rodents using a titanium alloy skinfold frame (SM100, APJ Trading Co., Ventura, USA) [47]. The DSWC mouse model is a well-established platform for investigating vascular responses during V-PDT [48,49], allowing for continuous, high-resolution visualization of microvascular structures. Although the therapeutic efficacy of V-PDT is conventionally evaluated several weeks post-treatment, the DSWC model permits immediate and intuitive assessment of vascular responses during and shortly after irradiation.

### V-PDT protocol

In the established DSWC rodent model, HMME was administered via tail vein injection at a dose of 10 mg/kg. V-PDT was initiated 1 min post-injection, with the total light dose fixed at 60 J/cm^2^ across all groups. Thirty mice were randomly assigned to 3 experimental groups (*n* = 10 per group), corresponding to 3 distinct irradiation protocols:

1. High-irradiance, short-duration protocol: continuous illumination at 100 mW/cm^2^ for 10 min.
2. Low-irradiance, long-duration protocol: continuous illumination at 25 mW/cm^2^ for 40 min.
3. Personalized-irradiation protocol: illumination initiated at 100 mW/cm^2^ and then dynamically modulated between 25 and 100 mW/cm^2^ according to real-time sO₂ feedback. The exposure duration was dynamically adjusted to ensure that the total delivered light dose remained 60 J/cm^2^.

### Oxygen saturation reconstruction

As illustrated in Fig. 7B(i), a spectral second-order derivative-based oxygen reconstruction algorithm was employed in this study to calculate tissue oxygen saturation profiles. Reflectance spectral images were acquired at 6 specific wavelengths: 544, 552, 568, 576, 592, and 600 nm [50]. The primary advantage of this algorithm lies in its ability to suppress interference from endogenous chromophores such as melanin in the skin, which could otherwise affect measurement accuracy. This is achieved by processing the raw tissue spectra into second-order derivatives, thereby enhancing the specificity of the oxygen saturation estimation. The acquisition time for each sO₂ map was approximately 0.9 s, providing sub-second temporal resolution for tracking oxygen saturation dynamics during PDT.

### Photosensitizer concentration reconstruction

To obtain accurate fluorescence intensity data of the photosensitizer, a compensation algorithm was employed to correct for fluorescence attenuation caused by the optical properties of biological tissues. As illustrated in Fig. 7B(ii), a dual-band normalization-based correction algorithm was utilized to achieve wide-field quantitative fluorescence imaging. This method does not require explicit light propagation modeling across the entire FOV. Instead, it derives a correction factor by integrating reflectance spectra from 2 distinct wavelength ranges, thereby simplifying the overall data processing workflow [51]. Subsequently, the acquired fluorescence signals were spectrally unmixed using a regression-based algorithm to eliminate interference from nonspecific signals and extract pure photosensitizer fluorescence. Finally, using precalibrated conversion parameters, the processed fluorescence signals were quantitatively converted into concentration data. For quantitative photosensitizer reconstruction, diffuse reflectance spectra at 520 to 540 nm and 600 to 620 nm were acquired once prior to PDT. During irradiation, only the fluorescence bands from 600 to 630 nm (4-nm increment) were collected, resulting in a photosensitizer concentration acquisition time of approximately 1.2 s per frame.

### Quantification of vascular damage

As shown in Fig. 7D, to quantitatively assess V-PDT-induced vascular damage, the PAM of the dorsal skinfold window before and after treatment was converted into binary images. The VSR (%) was then calculated based on the change in the number of white pixels between the 2 binary images. As shown in Eq. 1, the VSR is defined as:

$$\mathrm{VSR}=\frac{N_{\text{before}}-N_{\text{after}}}{N_{\text{before}}}$$ (1)

where *N*_before_ and *N*_after_ represent the number of foreground pixels (i.e., vessel area) in the binary PAM images before and after treatment, respectively.

### Quantitative metrics for oxygen dynamics

The oxygen depletion rate (*K*_sO₂_, %/min) was defined as the absolute average slope of the sO₂ time course from the beginning of irradiation to the moment when sO₂ first dropped to 30%:

$$K_{{\mathrm{sO}}_{2}}=\left|\frac{sO_{2}\left(t_{\text{start}}\right)-sO_{2}\left(t_{30\text{\%}}\right)}{t_{30\text{\%}}-t_{\text{start}}}\right|$$ (2)

where *t*_start_ denotes the onset of PDT illumination and *t*_30%_ is the time when sO₂ first dropped to 30%. This metric reflects the rate at which oxygen is consumed during PDT.

The time-to-hypoxia (*T*_hypoxia_, s) was defined as the elapsed time from irradiation onset to the moment when sO₂ first dropped below 30%:

$$T_{\text{hypoxia}}=t\left(sO_{2}<30\text{\%}\right)$$ (3)

where *t*(*sO₂* < 30%) denotes the time point when sO₂ first drops below 30%. A longer *T*_hypoxia_ indicates slower oxygen depletion and improved oxygen availability during V-PDT.

The oxygen recovery amplitude (*ΔsO₂*, %) was defined as the increase in sO₂ from the end of PDT to the value measured 300 s after treatment:

$$\Delta sO_{2}={sO_{2}}^{300s}-{sO_{2}}^{\mathrm{end}}$$ (4)

where *sO₂*^300s^ denotes the oxygen saturation measured 300 s after PDT, and *sO₂*^end^ denotes the oxygen saturation measured immediately at the end of PDT. This metric quantifies the extent of oxygen restoration following illumination and reflects tissue reoxygenation capacity.

### Statistical analysis

All statistical analyses were performed using GraphPad Prism 10. Data are presented as mean ± SD. Data normality was assessed using the Shapiro–Wilk test. Differences among the 3 treatment groups (high-irradiance, low-irradiance, and personalized-irradiation; *n* = 10 mice per group) were evaluated using one-way ANOVA. When a significant group effect was detected, Tukey’s honest significant difference (HSD) post hoc test was applied for pairwise comparisons. A *P* value of <0.05 was considered statistically significant.

## Data Availability

Data will be made available on request.
